# Apalutamide, Darolutamide and Enzalutamide for Nonmetastatic Castration-Resistant Prostate Cancer (nmCRPC): A Critical Review

**DOI:** 10.3390/cancers14071792

**Published:** 2022-03-31

**Authors:** Carlo Cattrini, Orazio Caffo, Ugo De Giorgi, Alessia Mennitto, Alessandra Gennari, David Olmos, Elena Castro

**Affiliations:** 1 Department of Medical Oncology, “Maggiore della Carità” University Hospital, 28100 Novara, Italy; carlo.cattrini@maggioreosp.novara.it (C.C.); mennitto.alessia@gmail.com (A.M.); alessandra.gennari@uniupo.it (A.G.); 2 Medical Oncology, Department of Translational Medicine (DIMET), University of Eastern Piedmont (UPO), 28100 Novara, Italy; 3 Department of Internal Medicine and Medical Specialties (DIMI), University of Genoa, 16132 Genoa, Italy; 4 Department of Medical Oncology, Santa Chiara Hospital, 38122 Trento, Italy; orazio.caffo@apss.tn.it; 5 Department of Oncology, IRCCS Istituto Romagnolo per lo Studio dei Tumori (IRST) “Dino Amadori”, 47014 Meldola, Italy; ugo.degiorgi@irst.emr.it; 6 Department of Medical Oncology, Hospital Universitario 12 de Octubre, Instituto de Investigación Sanitaria Hospital 12 de Octubre (imas12), 28041 Madrid, Spain; dolmos.imas12@h12o.es; 7 Genitourinary Cancer Translational Research Group, Instituto de Investigación Biomédica de Málaga, 29010 Málaga, Spain; 8 UGCI Medical Oncology, Hospitales Universitarios Virgen de la Victoria y Regional de Málaga, 29010 Málaga, Spain

**Keywords:** nonmetastatic castration-resistant prostate cancer, nmCRPC, androgen-receptor signaling inhibitors, conventional imaging, PSMA-PET, apalutamide, darolutamide, enzalutamide

## Abstract

**Simple Summary:**

Apalutamide, darolutamide and enzalutamide are androgen-receptor signaling inhibitors proved to be useful in patients with nonmetastatic castration-resistant prostate cancer (nmCRPC). Although computed tomography and bone scans have been used to identify patients with nmCRPC in pivotal trials, currently, novel imaging techniques are widely used to stage patients, and they can detect metastases in many men with nmCRPC who are negative to conventional imaging. This review aims at discussing the role of apalutamide, darolutamide and enzalutamide in nmCRPC and the clinical implications of novel imaging techniques during treatment choice.

**Abstract:**

Nonmetastatic castration-resistant prostate cancer (nmCRPC) represents a condition in which patients with prostate cancer show biochemical progression during treatment with androgen-deprivation therapy (ADT) without signs of radiographic progression according to conventional imaging. The SPARTAN, ARAMIS and PROSPER trials showed that apalutamide, darolutamide and enzalutamide, respectively, prolong metastasis-free survival (MFS) and overall survival (OS) of nmCRPC patients with a short PSA doubling time, and these antiandrogens have been recently introduced in clinical practice as a new standard of care. No direct comparison of these three agents has been conducted to support treatment choice. In addition, a significant proportion of nmCRPC on conventional imaging is classified as metastatic with new imaging modalities such as the prostate-specific membrane antigen positron emission tomography (PSMA-PET). Some experts posit that these “new metastatic” patients should be treated as mCRPC, resizing the impact of nmCRPC trials, whereas other authors suggest that they should be treated as nmCRPC patients, based on the design of pivotal trials. This review discusses the most convincing evidence regarding the use of novel antiandrogens in patients with nmCRPC and the implications of novel imaging techniques for treatment selection.

## 1. Introduction

Prostate cancer (PCa) is the most frequently diagnosed neoplasm and among the leading causes of cancer deaths in Western countries [1]. Approximately 15–30% of PCa patients who undergo either surgery or radiotherapy for PCa experience prostate-specific antigen (PSA) recurrence [2,3,4]. Many of these patients, together with men who show disease unsuitable for radical treatment, receive androgen-deprivation therapy (ADT). The duration of response to ADT can last from months to many years, and this disease stage is known as hormone-sensitive prostate cancer (HSPC). The long-term exposure to ADT eventually results in disease progression despite castration, a clinical condition that is defined as castration-resistant prostate cancer (CRPC). CRPC represents a potentially life-threating disease state that can be observed both in men with metastatic (m) and nonmetastatic (nm) CRPC who have received prior ADT. In the last two years, three landmark clinical trials have been published, and the treatment landscape of nmCRPC has radically changed. This comprehensive review focuses on the advances and concerns regarding the management of patients with nmCRPC. In this comprehensive review, we discuss the most convincing literature data on the role of novel antiandrogens for the treatment of patients with nmCRPC. We also highlight the risk of stage migration using novel imaging techniques and its clinical implications.

## 2. Nonmetastatic Castration-Resistant Prostate Cancer

NmCRPC is a condition characterized by biochemical progression during ADT, despite castrate serum testosterone <50 ng/dl or 1.7 nmol, without evidence of metastatic disease by conventional imaging (CIM) [5]. NmCRPC covers a broad spectrum of clinical scenarios, including patients with loco-regional PCa (relapse, residual disease or primary tumors) as well as men with rising PSA and undetectable disease after treatment with curative purpose. Patients with N1 regional lymph node metastases (nodes of the true pelvis, which are essentially the pelvic nodes below the bifurcation of the common iliac arteries) are included in nmCRPC. 

The Prostate Cancer Working Group 3 (PCWG3) consensus for metastatic PCa defines PSA progression as a ≥25% PSA increase with an absolute increase of ≥2 ng/mL from the nadir. This PSA rise must be confirmed by a second value obtained ≥3 weeks later, in the context of castrate testosterone values (<50 ng/dL). However, the pivotal trials described below used quite different criteria for biochemical progression and, therefore, to define their patients as nmCRPC. All trials required three consecutive rising PSA levels during ADT at least 1 week apart despite castrate level of serum testosterone, with the last PSA > 2 ng/mL. Additionally, SPARTAN and ARAMIS required that the PSA rise resulted in two > 50% PSA increases over nadir, whereas the PROSPER trial did not specify a percentage of increase. 

Before 2018, no standard of care was established for patients with nmCRPC progressing on ADT. Maximal androgen blockade (MAB), consisting of the addition of a first-generation antiandrogen (i.e., bicalutamide) to ADT, was often proposed to these patients.

This therapeutic approach was attempted based on results from patients with metastatic PCa, in which a slight survival benefit was observed by the use of immediate MAB compared to ADT alone [6]. However, the benefit in nonmetastatic patients was unclear and little evidence supported the role of deferred MAB after ADT progression in patients with nmCRPC [7,8]. After ADT progression in nmCRPC, first-generation antiandrogen monotherapy (i.e., bicalutamide), switching or withdrawal of antiandrogens also provided short-term PSA responses, but no clinical trial has ever demonstrated a survival benefit by using such approaches [9,10,11]. 

### 2.1. Lessons from a Denosumab Trial to Identify High-Risk nmCRPC Patients

In 2012, Smith et al. published the results of a large phase 3 trial that investigated the role of denosumab, a fully human anti-RANKL monoclonal antibody, for prevention of bone metastasis in men with nmCRPC [12]. Overall, 1432 patients were randomized to receive 120 mg denosumab or placebo every 4 weeks. The primary end point of increased bone-metastasis-free survival (bone-MFS) was met (4.2 months improvement, hazard ratio (HR) 0.85, 95% CI 0.73–0.98, *p* = 0.028). Denosumab also delayed time to first bone metastasis (HR 0.84, 95% CI 0.71–0.98, *p* = 0.032) and time to symptomatic bone metastasis (HR 0.67, 95% CI 0.49–0.92, *p* = 0.01). However, these benefits did not translate into an overall survival (OS) difference between groups (HR 1.01, 95% CI 0.85–1.20, *p* = 0.91). Importantly, a PSA ≥ 8 µg/L within 3 months before randomization or a PSA doubling time (PSA-dt) ≤ 10 months (or both) were identified as risk factors for developing bone metastases. A subgroup analysis in the placebo group also showed that median bone-MFS in men with PSADT < 6 months was 6.5 months shorter compared to the entire placebo population (18.7 months vs. 25.2 months) [13]. In patients with aggressive PSA kinetics, the effect of denosumab on bone-MFS was more pronounced, resulting in a median prolongation of 7.2 months compared to placebo (HR 0.77, 95% CI 0.64–0.93, *p* = 0.0064). Rates of adverse events were similar in both groups, except for osteonecrosis of the jaw (5%) and hypocalcemia that were more frequent in the investigational arm. 

Given that denosumab did not prolong OS and the limited improvement in terms of bone-MFS was not balanced by the potential side effects, the United States Food and Drug Administration (FDA) and the European Medicines Agency (EMA) did not approve this agent for the treatment of nmCRPC [14]. However, this trial provided important information about the relevance of PSA dynamics in patients with nmCRPC and put the basis for the subsequent design of clinical trials in this setting.

### 2.2. Landmark Clinical Trials in nmCRPC

The pressure of ADT prompts cancer cells to develop adaptive mechanisms of survival that cause the transition to castration-resistant disease. These processes can involve the androgen-receptor (AR) pathway but can also be AR-independent mechanisms [15,16]. Nonetheless, alterations in AR signaling are the most frequent biological events in CRPC, resulting in persisting AR activation. These alterations include AR amplifications, mutations, AR splice variants, intratumoral androgen synthesis and AR enhancer amplification, among others. Apalutamide, darolutamide and enzalutamide are new-generation antiandrogens that not only competitively inhibit the AR ligand-binding domain with higher affinity than first-generation agents, but they also impair AR translocation to the nucleus and obstruct AR-mediated transcription [17]. 

In April 2018, Smith and colleagues published the results of the phase 3 SPARTAN trial, which evaluated the efficacy of apalutamide in preventing metastasis occurrence in men with nmCRPC and had a PSA-dt ≤ 10 months [18]. Apalutamide is a novel antiandrogen with a chemical structure similar to enzalutamide. The in vivo experiments suggested that apalutamide might have greater antitumor activity and lower penetration into the blood–brain barrier than enzalutamide, and the phase 2 trial showed promising results in men with nmCRPC [19,20]. In the SPARTAN trial, patients with malignant pelvic lymph nodes that measured less than 2 cm in the short axis (classified as N1) and that were located below the aortic bifurcation were allowed to participate in this study. A total of 1207 men were randomly assigned to receive, in addition to ADT, apalutamide 240 mg per day or placebo. The primary end point was met, with a 2-year improvement on median metastasis-free survival (MFS) with apalutamide compared with placebo (40.5 vs. 16.2 months, HR 0.28, 95% CI 0.23–0.35, *p* < 0.001). Time to metastasis, progression-free survival (PFS), and time to symptomatic progression were also significantly longer in the experimental arm (*p* < 0.001 for all comparisons). At the first interim analysis with a median follow-up of 20.3 months, no statistically significant improvement in OS (secondary end point) was reached (HR 0.70, 95% CI: 0.47–1.04, *p* = 0.07). However, based on the achievement of the primary end point and the good safety-tolerability profile, the U.S. Food and Drug Administration (FDA) and the European Medicines Agency (EMA) granted approval for apalutamide in men with high-risk nmCRPC. The final analysis for OS at median follow-up of 52 months demonstrated a significant OS benefit from treatment with apalutamide plus ADT compared to ADT plus placebo (73.9 vs. 59.9 months, HR: 0.78, 95% CI: 0.64–0.96, *p* = 0.016), despite 19% crossover (placebo to apalutamide) and higher rates of subsequent therapy in the placebo group [21]. Apalutamide also prolonged time to cytotoxic chemotherapy compared to placebo (HR: 0.63, 95% CI: 0.49–0.81, *p* = 0.0002). In addition, apalutamide extended median progression-free survival 2 (PFS2) by 14.4 mo versus placebo (55.6 vs. 41.2 months) and reduced the hazard of second progression or death by 45% (HR: 0.55, 95% CI: 0.46–0.66, *p* < 0.0001). Discontinuation rates due to adverse events were low (15% and 8.4% in apalutamide and placebo arms). Of note, rash was the side effect with the greatest difference in incidence between the two arms (23.8% vs. 5.5%). (Table 1)

Two months after the publication of SPARTAN, Hussain and colleagues published the results of the phase 3 PROSPER study, which assessed the efficacy of enzalutamide in preventing metastasis development in men with nmCRPC and a PSA-dt ≤ 10 months [22]. The efficacy of enzalutamide in CRPC was well known at the time based on the phase 3 trials in pre- and post-docetaxel mCRPC [23,24]. The phase 2 STRIVE trial also provided promising results in nmCRPC [25]. Patients enrolled in the PROSPER trial had to have a baseline PSA level ≥ 2 ng/mL, and no evidence of nodal or distant metastases. A total of 1401 patients were randomized to receive enzalutamide 160 mg or placebo once daily in addition to ADT. At a median follow up of 16.8 months, the primary end point was met, with a median MFS of 36.6 months in the enzalutamide group versus 14.7 months in the placebo arm (HR 0.29, 95% CI, 0.24–0.35, *p* < 0.001). The time to the first use of a subsequent antineoplastic therapy and time to PSA progression were longer with enzalutamide than with placebo (*p* < 0.001 for both). At the first interim analysis of OS, 103 patients (11%) receiving enzalutamide and 62 (13%) receiving placebo had died. Based on the MFS advantage, the FDA and the EMA extended approval for enzalutamide for men with high-risk nmCRPC in addition to previous approval for mCRPC. The final OS analysis published in June 2020 showed a statistically significant benefit in patients treated with enzalutamide compared to placebo (67.0 vs. 56.3 months, HR: 0.73, 95% CI: 0.61 to 0.89, *p* = 0.001) [26]. Adverse events were also consistent with the established safety profile of enzalutamide (Table 1).

In February 2019, Fizazi and colleagues published the results of the phase 3 ARAMIS trial, which investigated the efficacy of darolutamide in preventing metastatic progression in men with nmCRPC and had a PSA-dt ≤ 10 months [27]. Darolutamide is another novel antiandrogen with a distinct chemical structure compared to both enzalutamide and apalutamide. It has been shown to be active in cells harboring the missense F876L mutation in the ligand-binding domain of the AR, which confers resistance to enzalutamide and apalutamide [28]. Notably, in vivo studies did not demonstrate an increase in serum testosterone levels by exposure to darolutamide, and this drug had negligible blood–brain barrier penetration [28]. Darolutamide showed significant antitumor activity and a good side effect profile in the phase 1–2 studies in men with mCRPC [29]. Similar to the SPARTAN trial, the presence of pelvic lymph nodes <2 cm in diameter in the short axis below the aortic bifurcation was not an exclusion criterion of the ARAMIS trial. Globally, 1509 patients underwent randomization in a 2:1 ratio and received, in addition to ADT, either darolutamide 1200 mg daily or placebo. At a median follow up of 17.9 months, darolutamide prolonged the median MFS compared to placebo (40.4 vs. 18.4 months, respectively, HR 0.41, 95% CI 0.34–0.50, *p* < 0.001). Darolutamide was also associated with benefits in all secondary end points, including OS, time to pain progression, time to cytotoxic chemotherapy and time to a symptomatic skeletal event. These results prompted the FDA to grant fast track designation for darolutamide that was finally approved for men with high-risk nmCRPC in July 2019. The final analysis, published in September 2020, demonstrated a clear OS advantage for patients treated with darolutamide (HR: 0.69, 95% CI: 0.53 to 0.88, *p* = 0.003) [30]. Fifty-five percent of patients originally assigned to placebo received subsequent life-prolonging therapy (31% crossed-over to darolutamide), compared to 15% of those who received darolutamide. Benefit with respect to all other secondary end points, including time to first symptomatic skeletal event and time to first use of cytotoxic chemotherapy, was also confirmed (Table 1).

**Table 1:** Phase 3 randomized clinical trials in nmCRPC.

		SPARTAN	PROSPER	ARAMIS
Antiandrogen		Apalutamide	Enzalutamide	Darolutamide
**STUDY DESIGN**	Inclusion criteria	- M0 N0–1 CRPC - PSA rising - PSAdt ≤ 10 mo - PSA ≥ 2 ng/mL	- M0 N0 CRPC - PSA rising - PSAdt ≤ 10 mo - PSA ≥ 2 ng/mL	- M0 N0–1 CRPC - PSA rising - PSAdt ≤ 10 mo - PSA ≥ 2 ng/mL
	Stratification factors	- PSA-dt > 6 vs. ≤6 mo - Prior use of bone-sparing agents - Nodal disease (N0 vs. N1)	- PSA-dt > 6 vs. ≤6 mo - Prior use of bone-sparing agents	- PSA-dt > 6 vs. ≤6 mo - Use of osteoclast-targeted therapy at randomization
	Primary endpoint	MFS, defined as time from randomization to the first detection of distant metastasis on imaging or death from any cause	MFS, defined as the time from randomization to radiographic progression, or death from any cause between randomization and 112 days after drug discontinuation without evidence of radiographic progression	MFS, defined as the time from randomization to confirmed evidence of distant metastasis on imaging or death from any cause
	Secondary endpoints	- Time to metastasis - PFS - Time to symptomatic progression - OS - Time to chemotherapy	- TTPP - PSA response rate - Time to use of new antineoplastic agent - Quality of life - OS - Safety	- OS - Time to pain progression - Time to first symptomatic SRE - Time to first cytotoxic therapy
**POPULATION**	Patients	Total randomized: *n* = 1207 Apalutamide + ADT (*n* = 806) vs. Placebo + ADT (*n* = 401)	Total randomized: *n* = 1401 Enzalutamide + ADT (*n* = 933) vs. Placebo + ADT (*n* = 468)	Total randomized: *n* = 1509 Darolutamide + ADT (*n* = 955) vs. Placebo + ADT (*n* = 554)
	Median PSA-dt	Experimental: 4.4 mo Placebo: 4.5 mo	Experimental: 3.8 mo Placebo: 3.6 mo	Experimental: 4.4 mo Placebo: 4.7 mo
	Nodes positivity	16.5% vs. 16.2% (placebo)	–	17% vs. 29% (placebo)
	Median FU	52 mo (interim 20.3 mo *)	48 mo (interim 16.8 mo *)	29 mo (interim 17.9 mo *)
**EFFICACY**	MFS	40.5 mo vs. 16.2 mo (placebo) HR 0.28 (95% CI 0.23–0.35), *p* < 0.001 *	36.6 mo vs. 14.7 mo (placebo) HR 0.29 (95% CI 0.24–0.35), *p* < 0.001 *	40.4 mo vs. 18.4 mo (placebo) HR 0.41 (95% CI, 0.34–0.50), *p* < 0.001 *
	TTPP	40.5 vs. 3.7 mo (placebo) HR 0.07 (95% CI 0.06–0.09)	37.2 mo vs. 3.9 mo (placebo) HR 0.07 (95% CI 0.05–0.08) *	33.2 mo vs. 7.3 mo (placebo) HR 0.13 (0.11–0.16) *
	PFS	40.5 mo vs. 14.7 mo (placebo) HR 0.29 (95% CI 0.24–0.36) *	Not reported	36.8 mo vs. 14.8 mo placebo HR 0.38 (0.32–0.45) *
	PFS2	55.6 mo vs. 41.2 mo (placebo) HR 0.55 (95% CI 0.46–0.66)	Not reported	Not reported
	Time to symptomatic progression	HR 0.57 (0.44–0.73) *p* < 0.0001 favoring apalutamide	Not reported	HR 0.65 (0.53–0.79) (pain progression)
	Time to first chemotherapy	HR 0.63 (0.49–0.81) favoring apalutamide	HR 0.54 (0.44–0.67) favoring enzalutamide	HR 0.58 (0.44–0.76) favoring darolutamide
	OS	73.9 vs. 59.9 mo (placebo) HR 0.78 (95% CI 0.64–0.96), *p* = 0.016	67.0 mo vs. 56.3 mo HR 0.73 (95% CI 0.61–0.89), *p* = 0.001	40.4 mo vs. 18.4 mo HR 0.69 (0.53–0.88), *p* = 0.003
**SAFETY**	Median duration of treatment	32.9 mo vs. 11.5 mo (placebo)	33.9 mo vs. 14.2 mo (placebo)	25.8 mo vs. 11.6 mo (placebo)
	AEs profile	SAEs: 36% vs. 25% (placebo); AEs leading to drug discontinuation: 15% vs. 7.3%; AEs with death 3% vs. 0.5%	SAEs: 40% vs. 22% (placebo); AEs leading to drug discontinuation: 17% vs. 9%; AEs with death 5% vs. 1%	SAEs: 26.1% vs. 21.8% (placebo); AEs leading to drug discontinuation: 8.9% vs. 8.7%; AEs with death 4% vs. 3.4%
	Most frequent ≥ 3 AEs	Hypertension: 16% vs. 12% Skin rash: 5.2% vs. 0.3% Fracture: 4.9% vs. 1.0% Falls: 2.7% vs. 0.8%	Hypertension: 6% vs. 2% Fatigue: 4% vs. 1% Major cardiovascular events: 4% vs. 2% *	Hypertension: 3.5% vs. 2.3% Coronary-artery disorders: 2% vs. 0.4% Cardiac arrhythmia: 1.8% vs. 0.7%
Bibliography		[18,21]	[22,26]	[27,30]

AEs: adverse events; FU: follow up; MFS: metastasis-free survival; mo: months; OS: overall survival; PD: progressive disease; PFS: progression-free survival; PSA-dt: prostate-specific antigen doubling time; SRE: skeletal-related event; TTPP: time to PSA progression; * Data from planned primary analysis.

### 2.3. Are There Still nmCRPC Patients?

**Figure 1:**
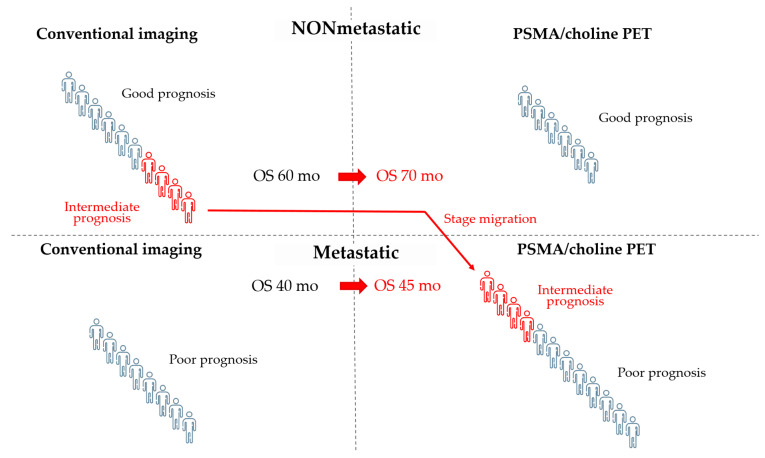
The use of PSMA or choline PET can cause stage migration and the *Will Rogers phenomenon*. In this example, patients who are metastatic by PET but not by conventional imaging (CIM) show an intermediate prognosis, which is better than that of metastatic patients and worse than that of nonmetastatic patients by CIM. Staging by PET moves these patients with intermediate prognosis to the group of patients with metastatic disease and poor prognosis. The final result is an improvement in the prognosis of both nonmetastatic and metastatic groups of patients by CIM without changing the individual prognosis of patients. It is unknown whether modifying the therapeutic approach in the subgroup of patients with intermediate prognosis could lead to an improvement in their outcome. OS: overall survival.

Patients enrolled in PROSPER, SPARTAN and ARAMIS trials were assessed as nonmetastatic by CIM, including bone scintigraphy plus computed tomography (CT) or magnetic resonance imaging (MRI). The proPSMA randomized trial confirmed that prostate-specific membrane antigen positron emission tomography (PSMA-PET) has greater sensitivity and accuracy compared to CIM with less radiation exposure [31], and the use of more accurate imaging tests, such as choline or PSMA-PET, results in a migration to metastatic stage in a significant number of patients classified as nmCRPC by CIM. A retrospective analysis in 1007 men with biochemically recurrent disease revealed that 68Ga-PSMA-11 PET can detect tumor lesions in almost 80% of patients [32]. In another retrospective study on 200 patients with nmCRPC, PSMA-PET was positive in 196 of 200 patients. Of these, 44% had pelvic diseases, including 24% with local prostate bed recurrence, and 55% had metastatic disease despite negative CIM [33]. Importantly, the prognosis of migrated patients would be worse than that of those who remained in the nonmetastatic stage, but better than that of patients classified as metastatic by CIM. As a result, the extensive use of PSMA-PET is likely to improve the prognosis of both groups, nmCRPC and mCRPC, without any change in individual outcomes. This effect is known as the *Will Rogers phenomenon* (Figure 1).

**Figure 2:**
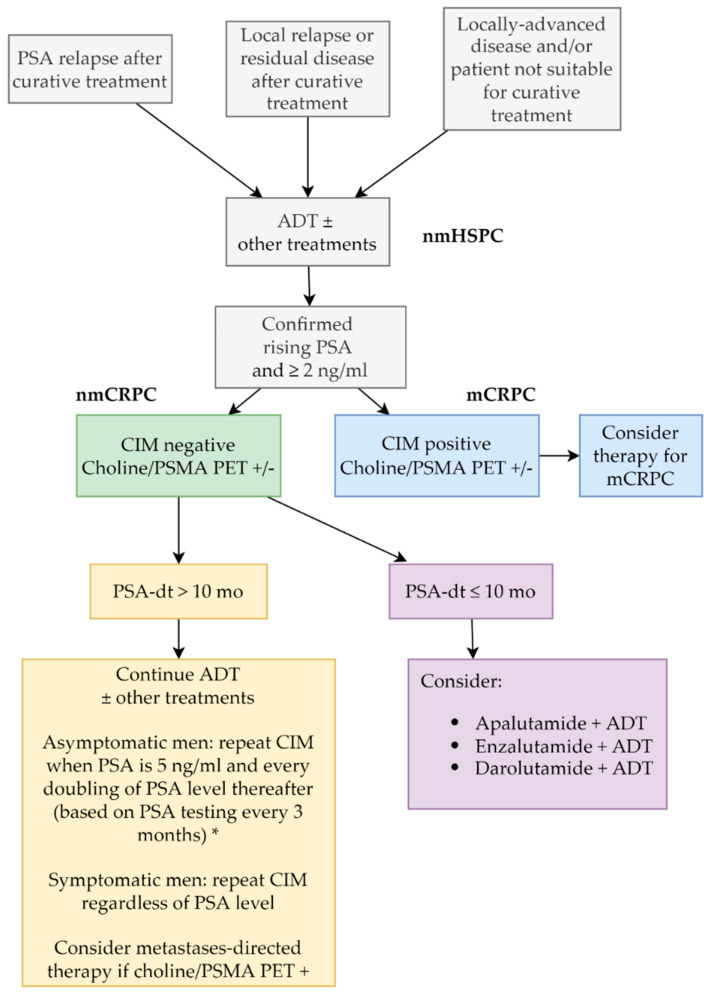
Proposed algorithm of treatment in patients with nmCRPC (this algorithm is a advice from the authors, not a guideline). * Consensus statement by the RADAR group [35]. ADT: androgen-deprivation therapy; CIM: conventional imaging; mCRPC: metastatic castration-resistant prostate cancer; nmCRPC: nonmetastatic castration-resistant prostate cancer; nmHSPC: nonmetastatic hormone-sensitive prostate cancer; PET: positron emission tomography; PSA: prostate-specific antigen; PSA-dt: PSA doubling time; PSMA: prostate-specific membrane antigen.

It is currently unclear whether treatment decisions in a CRPC setting should change based on new imaging techniques. Pivotal trials for nmCRPC used CIM for staging and, therefore, included a large proportion of patients that would be metastatic by PSMA-PET, even if they were negative to CIM as all trials in the nmCRPC scenario have demonstrated unequivocal benefit in these patients. On the other hand, the benefit of treating CIM-negative/ PET-positive patients as mCRPC has not been demonstrated, given that almost all randomized trials in the mCRPC scenario have used CIM as a standard method to assess the presence, size and diffusion of metastases. Even if PSMA-PET can be useful in the context of metastases-directed therapy, currently there is no evidence to offer treatment options that are not based on CIM, unless clinical trials demonstrate that staging according to novel imaging techniques can improve the outcome of patients. As discussed in the editorial by Sundahl and colleagues, “using more sensitive novel imaging may be like driving a Ferrari across London when a Mini will also get you there, but with less angst” [34]. The main risk of patients’ upstaging from nmCRPC to mCRPC is related to the restriction of drugs to specific settings. Neither apalutamide nor darolutamide can be used in mCRPC, and a therapeutic opportunity may be lost in patients who are immediately upstaged from nmCRPC to mCRPC. Our proposed algorithm to manage patients with nmCRPC is shown in Figure 2.

### 2.4. Intensification of Hormone Therapy in nmCRPC: Is Potential Toxicity Worth the Risk?

In terms of clinical outcome, the results of SPARTAN, ARAMIS and PROSPER trials support the general notion that an early intensified-treatment strategy is better than later therapy in men with PCa [36]. Many studies support this assumption in different settings. The phase 3 TOAD study demonstrated a significant survival benefit by immediate receipt of ADT versus deferred intervention in the setting of PSA-relapsed HSPC [37]. Treatment intensification with chemo-hormonal therapy and/or novel AR signaling inhibitors was established as a standard of care for patients with mHSPC [38]. The PEACE-1 trial recently demonstrated the superiority of the triplet including ADT plus chemotherapy plus abiraterone acetate in men with high-volume mHSPC [39]. In the same population, the ARASENS trial has demonstrated the benefit of adding darolutamide to ADT plus docetaxel. In high-risk localized disease the STAMEDE trial has demonstrated the benefit of two years of abiraterone in addition to ADT and radiotherapy to the primary tumor [40]. Overall, these data suggest that treatment intensification, as opposed to treatment waiting, can provide significant benefit for patients with both hormone-sensitive and castration-resistant disease.

The primary endpoints of the SPARTAN, PROSPER and ARAMIS trials were common, and apalutamide was the first drug to be approved by the FDA on the sole basis of improved MFS [41], followed by enzalutamide and darolutamide. The regulatory authorities considered that these novel hormonal agents had an acceptable safety and tolerability profile in their phase 3 trials, with a predicted improvement in OS and a favorable risk–benefit ratio. MFS has been shown to be a surrogate for OS in patients with localized PCa [42], and the final positive OS results of all these phase 3 trials confirmed that MFS could be a good surrogate for OS, similar to that observed in mCRPC in respect to PFS and second PFS [43].

Apalutamide, darolutamide and enzalutamide showed good tolerance and maintenance of quality of life (QoL). In the SPARTAN trial, higher rates of fatigue, rash, weight loss, arthralgia, falls, hypothyroidism and fractures were observed with apalutamide than with placebo [18]. However, apalutamide maintained favorable QoL, did not worse fatigue and the majority of patients reported being “not at all bothered” by side effects [44]. A sub-analysis also showed a good QoL profile in the older population [45]. In the PROSPER trial, more rates of fatigue, hot flushes, hypertension, falls, major adverse cardiovascular events and mental impairment disorders were reported with enzalutamide compared to placebo. In addition, a 15% mortality rate without documented progression was initially observed with enzalutamide, compared to only 2% in the placebo arm [22]. However, enzalutamide also showed a good profile in terms of decline in QoL, and prolonged urinary and bowel symptom control [46]. Furthermore, safety was consistent across age and regional subgroups [47]. The ARAMIS trial reported only slightly higher rates of fatigue, asthenia and rash, and no increased incidence of seizures, falls, fractures, cognitive disorder or hypertension with darolutamide compared to placebo [27]. This antiandrogen maintained QoL and significant delayed urinary and bowel symptoms [48]. In addition, darolutamide remained well tolerated at an extended follow up; almost all patients received the full planned dose during the trial, and almost all those with dose modifications were able to resume and re-establish the planned dose [49].

No direct comparisons among these novel antiandrogens are currently available, and it is therefore difficult to ascertain the best tolerable drug among apalutamide, darolutamide and enzalutamide. Of importance, the three pivotal trials had significant differences in adverse events reporting and in absolute adverse events risks between placebo arms; thus, it would be inappropriate to draw conclusions about the putative superiority of a specific drug based on the published results [50].

### 2.5. Other Possible Treatments for nmCRPC

As previously discussed, several studies have demonstrated the benefit of early treatment with new hormonal therapies supporting the investigation of agents approved for the treatment of advanced PC in the nonmetastatic scenario. Abiraterone acetate is currently approved for the treatment of mHSPC and mCRPC both in pre- and post-docetaxel settings [51,52]. The IMAAGEN phase 2 trial investigated abiraterone acetate plus prednisone in nmCRPC patients with PSA ≥ 10 ng/mL or PSA-dt ≤ 10 months [53]. Of the 122 evaluable patients, 106 (86.9%) achieved a 50% reduction in PSA and median time to radiographic progression was estimated at 41.4 months. Notably, 57 patients (43.5%) experienced serious adverse events, mainly hypertension (23.7%) and hypokalemia (6.9%), and 7 deaths were reported. No subsequent phase 3 trial with abiraterone acetate in the nmCRPC population has been conducted. Docetaxel was established in 2004 as a new standard of care for patients with mCRPC [54], but to our knowledge, no randomized trial has investigated the role of this drug in nmCRPC. The publication of the NRG Oncology RTOG 0521 trial has overturned the previous notion that chemotherapy shows limited efficacy in nonmetastatic PCa. This trial assessed the role of adjuvant docetaxel in 612 men with high-risk localized PCa (84% of patients had a Gleason score ≥ 8) and demonstrated an improvement of OS from 89% to 93% at 4 years (HR 0.69, 90% CI 0.49–0.97, *p* = 0.034) by addition of docetaxel to radiotherapy and ADT [55]. Patients treated with adjuvant docetaxel also showed improvement in disease-free survival and reduction in the six-year rate of distant metastasis. Notably, PSA failure rates were not significantly different between the arms, suggesting that docetaxel activity may focus on the androgen-insensitive clones that produce less PSA [55]. In addition, in the nmCRPC setting, a retrospective Japanese study showed that docetaxel use was associated with favorable prognosis [56]. Despite these encouraging results, the balance between clinical advantages and potential toxicity is unfavorable, limiting the use of docetaxel in early PC stages to selected cases. In addition, the novel antiandrogens show a more attractive safety and tolerability profile compared to chemotherapy in nonmetastatic patients. However, docetaxel shows a distinct mechanism of action that might prevent the lineage plasticity induced by AR inhibition and might represent a potential opportunity once new biomarkers are able to identify patients with intrinsic resistance to hormone therapy [57].

### 2.6. Biomarkers of Response to AR-Directed Therapies in the nmCRPC Scenario

In order to avoid overtreatment and unnecessary toxicities, it is essential to correctly identify patients with nmCRPC who require intensification of hormone treatment and those who are unlikely to respond to these agents. Data reported up to date demonstrate that the outcomes of nmCRPC patients are strictly associated with PSA-dt, and those with a short PSA-dt are at higher risk of metastatic progression and death [13]. Despite the lack of prognostic nomograms in the nmCRPC setting, other prognostic parameters have been identified in patients with PCa and might also be investigated in nmCRPC. As an example, Gleason score, together with PSA kinetics, are the major determinants of prognosis in patients with biochemical recurrence after radical treatment [58].

To investigate potential prognostic biomarkers that may serve to guide treatment intensification in the nmCRPC scenario, Feng et al. stratified patients included in the SPARTAN study into high-risk and low-risk categories for developing metastases based on a genomic classifier (GC) scores for high (GC > 0.6) and low to average (GC ≤ 0.6) and into basal and luminal subtypes [59]. All molecular subtypes benefited from treatment with apalutamide, particularly those patients with high GC and luminal subtypes, who showed the greatest improvement in MFS. Of note, basal tumors with high T-cell proliferation showed benefit similar to luminal tumors; moreover, transcriptional signatures of increased immune activity, decreased vascularization and reduced proliferative capacity were associated with long-term response to apalutamide [60]. 

The presence of androgen receptor splice variant 7 (AR-V7) has been associated with worse prognosis in patients with mCRPC receiving enzalutamide and abiraterone, and chemotherapy appears to be superior to AR-directed therapy in AR-V7-positive men [61,62,63]. Detection of AR-V7 was increased after treatment with androgen receptor signaling inhibitors and rare in patients progressing on sole ADT [64,65]. The prevalence of AR-V7 in nmCRPC patients is unknown, although likely to be low in patients not previously exposed to AR-directed therapy. Therefore, the potential role of AR-V7 for therapy selection in the nmCRPC scenario is currently unknown.

DNA damage repair (DDR) defects have been described in up to 10% and 27% of localized and metastatic prostate cancer, respectively [66,67,68], with unknown prevalence in the nmCRPC setting. No conclusive data are available regarding the potential use of DDR alterations for selecting the most appropriate management of prostate cancer patients beyond the use of poly ADP ribose-polymerase (PARP) inhibitors. Data reported on the prognostic implications of somatic DDR are inconsistent, excepting *CDK12* biallelic inactivation, which has been associated with rapid progression on ADT- and AR-targeted therapy in mHSPC and mCRPC, respectively. Germline *BRCA2* mutations are associated with poor clinical outcomes in localized and metastatic prostate cancer [69,70,71], but their role in nmCRPC has not been explored. However, considering the aggressiveness of the disease linked to germline *BRCA2* mutation, intensified therapy with an AR-targeted therapy may be a good option in nmCRPC *BRCA2* mutation carriers. 

### 2.7. Not All That Glitters Is Gold

Despite the exciting results of the SPARTAN, PROSPER and ARAMIS trials, the use of novel antiandrogens in nonmetastatic PCa might represent a double-edged sword. Approximately 30% reduced risk of death is proved by use of these agents, and additional benefits in secondary end points have already been demonstrated. However, several concerns arise due to the potential earlier occurrence of treatment resistance that can affect the availability of therapeutic options after progression to an AR-directed therapy in the metastatic CRPC scenario [57]. 

Retrospective data from patients with mCRPC suggest the possibility of cross-resistance among different AR signaling inhibitors and between hormone agents and chemotherapy as well as reduced activity when agents are used in sequence [72,73,74]. The praecox and long-term use of potent AR inhibitors can induce adaptive phenotypes in cancer cells through the activation of both AR-dependent and AR-independent survival pathways [15,16]. Histological dedifferentiation and lineage alterations, such as treatment-induced neuroendocrine prostate cancer (t-NEPC) and treatment-induced epithelial-to-mesenchymal transition (t-EMT), can result in rapid disease progression and resistance to both hormone agents and chemotherapy [75]. A study confirmed that use of abiraterone acetate and enzalutamide increases the percentage of t-NEPC, which are found in 17% of metastatic biopsies obtained from patients with mCRPC [76]. This aggressive form of PCa is associated with shortened survival and shows near-mutual exclusivity with the presence of DNA repair mutations [76]. Strategies to prevent the treatment-induced lineage crisis might include rapid drug cycling with collateral sensitivity, innovative drug combinations, intermittent therapy and bipolar androgen therapy [57,77,78]. The phase 2 PRINT clinical trial is ongoing to assess the feasibility for cycling therapies to prevent resistance in patients with mCRPC [79,80]. This study might provide valuable data to test this approach also in the setting of nmCRPC.

## 3. Conclusions

The SPARTAN, PROSPER and ARAMIS trials have shown that apalutamide, enzalutamide and darolutamide significantly reduce the risk of metastatic progression and death in patients with nmCRPC without worsening their QoL. These drugs should be offered as valid options to patients who do not show contraindications to receiving them. Biomarker studies are ongoing to identify patients who can derive the greatest benefit from these antiandrogens. The evidence reported to date does not allow definitive conclusions about the superior benefit or safety of one agent over another. Choline and PSMA-PET show greater accuracy in detecting metastases compared to CIM; however, stage migration caused by novel imaging techniques can result in significant patients’ over- and undertreatment. Further studies are needed to address the role of novel imaging techniques in stratifying patients for treatment in nmCRPC and mCRPC settings as well as to learn whether early long-term use of AR-targeting therapies may affect the biology of the disease and the response to subsequent treatments.
